# Exploring swallowing, feeding and communication characteristics of toddlers with severe acute malnutrition

**DOI:** 10.4102/sajcd.v69i1.874

**Published:** 2022-10-31

**Authors:** Casey J. Eslick, Esedra Krüger, Alta Kritzinger

**Affiliations:** 1 Department of Speech-Language Pathology and Audiology, Faculty of Humanities, University of Pretoria, Pretoria, South Africa

**Keywords:** communication difficulties, early identification, oral-sensorimotor dysfunction, oropharyngeal dysphagia, severe acute malnutrition, speech-language therapist, swallowing and feeding characteristics, toddler

## Abstract

**Background:**

Severe acute malnutrition (SAM) is associated with cognitive and motor deficits. Little is known about the swallowing, feeding and communication characteristics of hospitalised toddlers with SAM, limiting the abilities of speech-language therapists to provide effective early intervention.

**Objective:**

To explore the background, swallowing, feeding and communication characteristics of toddlers with SAM during in-patient nutritional rehabilitation.

**Method:**

An exploratory, prospective, collective case-study was conducted with three hospitalised toddlers who were 12–18 months old and independently diagnosed with SAM, at least 1 week after transitioning to oral feeding. Detailed case histories were compiled through medical file perusal and parent interviews. Cross-sectional clinical bedside assessments were completed with the Rossetti Infant-Toddler Language Scale and Schedule for Oral-Motor Assessment.

**Results:**

All three participants had a history of feeding difficulties before admission. Despite intact pharyngeal swallows, heterogeneous oral-sensorimotor dysfunction and disruptive feeding behaviours were identified. Risk for oropharyngeal dysphagia indicates the need to modify dietary consistencies to prevent prolonging recovery or SAM relapse. Participants had mild-to-moderate language delays, particularly in interaction-attachment, play and language comprehension, with an atypical moderate receptive and mild expressive language delay profile. None of the participants were referred for speech-language therapy.

**Conclusion:**

This exploratory research showed the oral-sensorimotor skills, swallowing and communication characteristics of children with SAM. Speech-language therapists could address oral-sensorimotor functioning, feeding difficulties and communication interaction delays before discharge to community-based management for SAM. Further investigation with a larger sample size is recommended.

**Contribution:**

Novel description of the oral-sensorimotor skills for feeding and the communication development of three severely malnourished toddlers with HIV and tuberculosis co-infection was presented. The complexity of the three cases is highlighted.

## Introduction

The pathophysiological response to severe acute malnutrition (SAM) – the most severe form of undernutrition – reduces immunity, impairs growth and delays neurodevelopment, undermining children’s educational and economic potential (Iannotti et al., 2016; World Health Organization [WHO] & United Nations Children’s Fund [UNICEF], 2009). As a consequence of severe nutritional deficiency within a short period, structural brain changes disrupt neuro-cognitive development (Bhargava & Singh, 2020). Research in sub-Saharan Africa identified delayed development of executive functioning, learning memory, motor and communication skills (Prado & Dewey, 2014; Ruiseñor-Escudero et al., 2016; Sudfeld et al., 2015). Timely medical management of SAM according to WHO 10-step guidelines reduced case fatality rates in South Africa from 30 to 40% to less than 15% (Ashworth, Khanum, Jackson, & Schofield, 2003; WHO, 2013). However, survivors experience long-lasting developmental delay, which has implications for rehabilitation services (Iannotti et al., 2016). Although motor and communication delays are reported (East et al., 2019), it is not known how swallowing, oral-sensorimotor function and language development are affected by SAM in young children. Further detailed description is needed to inform speech-language therapists (SLTs) service delivery for this vulnerable population.

Severe acute malnutrition is a preventable condition defined as a weight-for-height *z*-score (WHZ) below three standard deviations, mid-upper arm circumference less than 11.5 cm and/or presence of bilateral pitting oedema (WHO, 2013). Commonly characterised by delayed growth, low weight, muscle wastage, poor appetite, recurrent illness, fatigue, vomiting, diarrhoea, dehydration and irritability in children (WHO, 2013), SAM is the result of multiple cascading biological and psychosocial risk factors. Akombi et al. (2017) summarised the risk factors including male gender, low birth weight and size, diarrhoea and illness, non-exclusive or short duration breastfeeding (BF) and young maternal age. There is, however, little research available regarding maternal pregnancy, maternal health and birth history of young children with SAM.

Multisectoral causal factors at the household level are strongly associated with intergenerational poverty (Itaka & Omole, 2020; WHO & UNICEF, 2009). South African research identifies poor individual health, inappropriate feeding practices, poor access to healthcare, water, hygiene and sanitation services, inadequate maternal education and low socio-economic factors as risks for underweight-for-age in children (Chopra, 2003; Lesiapeto, Smuts, Hanekom, Du Plessis, & Faber, 2010). Furthermore, the coronavirus disease 2019 (COVID-19) pandemic increased unemployment rates, reduced household income, heightened food insecurity and disrupted essential routine healthcare services (Nyashanu, Simbanegavi, & Gibson, 2020). Consequently, aggravated risk factors for undernutrition exacerbate challenges to address poor maternal and child health, communicable disease epidemics such as human immunodeficiency virus (HIV) and tuberculosis (TB), high prevalence of non-communicable diseases and trauma or injury (Hofman & Madhi, 2020). These factors are recognised as the quadruple burden of disease, which characterise the South African healthcare context.

As both causes and consequences of malnutrition, a high prevalence of HIV and TB co-infection challenges the systematic treatment of SAM and underlying conditions. A high HIV prevalence of 40–50% is reported among children treated for SAM in South Africa (De Maayer & Saloojee, 2011; Muzigaba, Sartorius, Puoane, Van Wyk, & Sanders, 2017). The majority of children only receive an HIV and/or TB diagnosis after admission for SAM, and the similarities of symptomatic presentation between HIV, TB and SAM challenge treatment initiation (De Maayer & Saloojee, 2011; Muzigaba et al., 2017). This delay is concerning because delayed initiation of highly active antiretroviral therapy (HAART) anti-TB medication can cause and/or exacerbate malnutrition (WHO, 2013). Additionally, children with HIV or HIV exposure are shown to be at a higher risk of morbidity and mortality (De Maayer & Saloojee, 2011). Furthermore, nutritional rehabilitation progress is shown to be impacted by HIV status and HIV disease stage (Muzigaba et al., 2017). Similar to the sequelae of SAM, HIV poses a high risk of neurodevelopmental compromise, affecting physical growth, gross and fine motor, social and language domains in young children (Gómez et al., 2018; Ndiaye et al., 2021). Severe acute malnutrition, HIV and TB co-infection can increase the risk of mortality (De Maayer & Saloojee, 2011).

Interaction of biological factors within the child and inappropriate feeding practices by the caregiver, play a role in SAM, which may consequently delay maturation of feeding skills and increase the risk for oropharyngeal dysphagia (OPD). Early BF cessation and introduction of solids before 6 months, as well as insufficient quantities of food are associated with SAM in South Africa (Itaka & Omole, 2020). These poor feeding practices, in addition to inadequate dietary variety (food types and consistencies), indicate children’s environment may lack adequate oral-sensorimotor experiences for maturation of feeding and swallowing skills (Arvedson, Brodsky, & Lefton-Greif, 2020). Respiratory rate changes in respiration-related illnesses such as bronchiolitis or TB, which commonly co-occur with SAM, can further increase the risk of OPD (Barbosa, Gomes, & Fischer, 2014). Furthermore, motor delays associated with SAM may contribute to oral-sensorimotor dysfunction, thereby disrupting co-ordination of the swallowing sequence, compromising safety and efficiency and resulting in OPD (Dodrill & Gosa, 2015).

Speech-language therapists play a central role in early in-patient management of swallowing and feeding difficulties to address possible OPD when transitioning to oral feeds (Arvedson et al., 2020; Barbosa et al., 2014). Speech-language therapists may therefore support the SAM management team to prevent OPD exacerbating challenges to achieve nutritional goals, shorten hospital stay and reduce the risk of relapse. Children with SAM are known to present as hypotonic, weak and lacking stamina during acute recovery (Arvedson et al., 2020). However, there is a paucity of research on the swallowing and feeding characteristics of young children with SAM during in-patient nutritional rehabilitation.

The updated WHO treatment protocol for SAM (WHO, 2013) calls for community-based management. In South Africa, implementation of this SAM management protocol means that out-patient toddlers with SAM may have limited access to SLT services, which are centralised at hospitals (Nyashanu et al., 2020). Hospital-based SLTs, therefore, require an in-depth understanding of the oral feeding and communication characteristics of toddlers with SAM to render holistic services to affected families before discharge in areas where SLT services are scarce. The following research question was proposed: What are the characteristics of the backgrounds, swallowing, feeding and communication of three toddlers with SAM during in-patient nutritional rehabilitation?

## Research method and design

### Aim and design

An exploratory, prospective, collective case-study design was used to describe the backgrounds, swallowing, feeding and communication characteristics of three toddlers with SAM during in-patient nutritional rehabilitation. Cross-sectional, quantitative, structured parent interviews and participant assessments are described for three cases.

### Setting

Bedside assessments were conducted in the paediatric medical ward of a tertiary hospital in June 2021. Along with doctors and nursing staff, a paediatric multidisciplinary team (MDT) is available including SLTs, audiologists, dieticians, physiotherapists, occupational therapists, social workers and psychologists. Rehabilitation services before discharge include transitioning toddlers to Ready-to-Use Therapeutic Food (RUTF) and modified family meals, co-ordination of a stimulation programme, structured play by an occupational therapist and/or physiotherapist, and involving the mother in feeding, childcare, and stimulation activities as recommended by guidelines (Ashworth et al., 2003; Department of Health [DOH], n.d.).

### Participants

The researcher visited the paediatric ward of the hospital, communicated and confirmed with the referred dietician to determine potential participants for the study. Cases were utilised as presented at the time of investigation. Three toddlers (mean age 1 year 4 months) with SAM, independently diagnosed by paediatricians according to the WHO’s definition of the condition, were selected purposively (WHO & UNICEF, 2009). All three toddlers were in the rehabilitation phase – Steps 8 to 10 of the WHO standard 10-step SAM management guidelines (see Online Appendix 1; WHO, 2013), feeding orally for at least 1 week, with weight gain and discharge preparation being the priority. The managing paediatrician and dietician confirmed toddlers were medically fit to participate, meaning that toddlers were not in the acute phase and had moved to the rehabilitation phase of SAM recovery (WHO, 2013) where there was no longer life-threatening risk. Mothers of toddlers required English language proficiency to answer background information questions.

Characteristics of the participants are shown in Table 1, including that participants had normal birth weight and were each diagnosed with HIV and TB co-infection. HIV infection was confirmed by medical practitioners using HIV PCR tests. All three participants were established as seropositive and received HAART after their initial hospital admissions. In the toddlers’ Road to Health Booklets, no notes indicated the prescription of nevirapine which is used to prevent mother-to-child transmission of HIV during BF. Medical practitioners diagnosed TB using tuberculin skin tests and induced sputum or gastric washings for TB microscopy. According to participants’ medical files, based on parent report, all participants presented with delayed developmental milestones (Table 1). Clinical care and treatment procedures followed the WHO standard 10-step guidelines for management of SAM (Ashworth et al., 2003; WHO, 2013) (Online Appendix 1). Feeding progressed with F75 formula, a therapeutic milk product low in protein and sodium and high in carbohydrates used during initial management of SAM. At the time of data collection, all participants were drinking F100 formula by cup and were being prepared for discharge, including receiving frequent physiotherapy sessions. All participants were recently referred to occupational therapy and intervention was because pf begin the next week. Speech-language therapist assessment was not conducted by hospital staff.

**TABLE 1:** Toddler characteristics per case (*n* = 3).

Characteristic	Case 1	Case 2	Case 3
**Gender**	Female	Female	Female
**Current age**	1 year 3 months	1 year 6 months	1 year 2 months
**Birth order**	First	Second	Second
**Birth**			
Birth weight	3.03 kg	3.60 kg	3.04 kg
Gestational age	38 weeks	35 weeks	38 weeks
APGAR score (1 min; 10 min)	7/10; 9/10	7/10; 10/10	9/10; 10/10
**Problems with birth**			
PROM	Yes	No	No
PET	No	Yes	No
**Problems after birth**			
Hyperbilirubinemia	Yes (Phototherapy – 2 days)	No	No
Prior hospitalisation	Yes (twice)	No	No
Reason prior admission	(1) Dehydration (2) Blood transfusion, zinc supplement	N/A	N/A
Update immunisations	No	Yes	No
**Diagnoses**			
SAM	Yes	Yes	Yes
Stunting	Yes	No	Yes
HIV	Infected	Infected	Infected
TB	Infected	Infected	Infected
**Treatments**			
Anti-retroviral therapy	Since admission	Since admission	Since admission
TB medication	Since admission	Since admission	Since admission
**Other conditions**			
Recurrent diarrhoea	Yes	Yes	Yes
Vomiting	Yes	Yes	Yes
Recurrent cough	Yes	Yes	Yes
Recurrent ear infections	Yes	No	Yes
Seizures	No	Yes	Yes
**Milestones**			
Sit (without assistance)	Not achieved	7 months	8 months
Crawl	9 months	9 months	9 months
Walk (without assistance)	Not achieved	14 months	Not achieved
First word	Not achieved – Babbles	14 months	14 months
**Intervention**			
MDT	Paediatrician, Dietician, Social worker, Physiotherapist	Paediatrician, Dietician, Social worker, Physiotherapist	Paediatrician, Dietician, Social worker, Physiotherapist
Referral to SLT	No	No	No

APGAR, appearance, pulse, grimace, activity, respiration; SAM, severe acute malnutrition; PROM, Premature rupture of membranes; PET, Pre-eclamptic toxaemia; HIV, human immunodeficiency virus; MDT, Multidisciplinary team; SLT, speech-language therapist; TB, tuberculosis.

The three mothers were HIV positive, two of which defaulted ARV treatment during pregnancy and are also TB co-infected (Table 2). Participant 3’s mother reported attending three antenatal visits. None of the mothers attended the prescribed number of antenatal appointments. Participants 1 and 3 were born in April 2020 during South Africa’s highest COVID-19 lockdown measures. All mothers expressed they had sufficient support during pregnancy as well as to fulfil current child care responsibilities.

**TABLE 2:** Maternal characteristics per case (*n* = 3).

Characteristic	Case 1	Case 2	Case 3
**Current age (years)**	21	33	32
**Languages**			
First language	Sesotho	Xitsonga	Sepedi
Additional languages	English, Sepedi	English, Sepedi, Venda	English
**Marital status**	Never married	Never married	Married (traditional)
**Children**			
Number living children	1	2	2
Lost unborn baby	No	Yes	No
**Diagnoses**			
HIV	Positive	Positive	Positive
TB	No	Infected with TB while pregnant	Infected with TB while pregnant
**Treatments**			
Anti-retroviral	Yes	Defaulted in pregnancy *Restarted at baby birth*	Defaulted in pregnancy *Restarted at baby birth*
TB medication	N/A	No	No

HIV, human immunodeficiency virus; TB, tuberculosis; N/A, not applicable.

All participants reside with their mothers in informal houses with varying socio-economic status-related characteristics (Table 3). Despite monthly income levels above the lower-bound poverty line (PL), all mothers expressed experiencing regular food insecurity, which impacts their feeding practices. The mothers reported no substance use (smoking, alcohol or drugs) in their households.

**TABLE 3:** Socio-economic status-related characteristics (*n* = 3).

Characteristic	Case 1	Case 2	Case 3
**Housing status**			
Housing	Informal housing	Informal housing	Living with others -Informal housing
Number of people in household	3	3	5
**SES categorisations**			
Self-estimated SES category	Middle	High	Middle
Income category	Low	Middle	Low
Income	R3000	R10 000	R3250
Income according to PL	Upper-bound PL	Above PL	Lower-bound PL
Income per person	R1000	R3333	R650
**Mother**			
Years educated	13	3	15
Highest education†	Grade 12	No formal schooling	Grade 12
Employed	No	No	Yes
Literacy	Yes	Yes	Yes
**Father**			
Years educated	N/A – 12	13	13
Highest education†	N/A – Grade 11	Grade 12	Grade 12
Employed	N/A – Yes	Yes	Yes
Literacy	N/A – Yes	Yes	Yes
**Household health**			
Health conditions	HIV	HIV and TB	HIV and TB
**Utility access**			
Electricity	No	Yes	Yes
Water	Public tap	Public tap	Tap in home
Sanitation	Open toilet in home	Outhouse	Outhouse
**Healthcare access**			
Distance to nearest clinic	12 km	6 km	5 km
Time to nearest clinic	16 min	18 min	15 min
Cellphone	Yes	Yes	Yes
Consult traditional healer	Yes	Yes	Yes
Transport	Public/Friend’s car	Public	Public

SES, socio-economic status; PL, poverty line; TB, tuberculosis; N/A, not applicable.

† , Highest level of schooling completed.

### Data collection materials and procedures

#### Background information sheet

A structured background information sheet was used to extract relevant information as quantitative data, from medical file perusal and thereafter, parent interviews. Compiled questions were adapted from Fuls (2019), Kritzinger (2012) and Van der Linde, Swanepoel, Glascoe, Louw and Vinck (2015), relating to demographics, SES, early developmental factors as well as feeding practices. Further considerations of disruptive feeding behaviours were inferred from Arvedson et al. (2020). Mothers were shown a picture chart and asked to point out if they consider their SES to be low, middle or high, and to disclose their actual household income which was interpreted according to the 2021 National Food Poverty Lines. Detailed social work notes in medical files were used to corroborate social and demographic information.

#### The schedule for oral motor assessment

The schedule for oral motor assessment (SOMA) (Reilly, Skuse, Mathisen, & Wolke, 1995) was used. The tool has been shown to successfully assess oral-sensorimotor functioning across a variety of consistencies with South African infants (Fuls, Krüger, & Van der Linde, 2020; Lalbahadur, 2018). Strong content validity, adequate construct and predictive validity, intra-rater and test-retest reliability, with 85% sensitivity have shown that the SOMA is an appropriate tool to assess oral-sensorimotor functioning for feeding in children who are eight to 24 months old (Barton, Bickell, & Fucile, 2018; Reilly et al., 1995). Since publication of the SOMA, which refers to oral-motor functioning, the term oral-sensorimotor functioning is used to holistically describe motor co-ordination and sensory integration required for feeding (Arvedson et al., 2020).

Data were collected by an SLT with experience in assessment of swallowing and feeding of young children. Face-to-face ratings were used to assess feeding and swallowing skills, specifically, oral-sensorimotor function related to voluntary oral preparation – duration of bolus formation, bolus manipulation and oral transit, as well as initiation of pharyngeal swallow through tongue base propulsion. The following consistencies were rated using the food items specified: Liquid (bottle, sippy-cup and/or open cup) – breastmilk, formula or water; Puree – yoghurt; semi-solid – soft porridge; solid – butternut cubes; and cracker – ginger biscuits or *Nuttikrust* biscuits. This research used the same food items for the SOMA as recommended by Fuls et al. (2020), who adapted ‘unknown foods’ from the SOMA, to foods recognised in the South African setting. Oral-sensorimotor functions such as upper and lower lip involvement for food removal, lip closure around food for removal and to prevent food loss, tongue protrusion, jaw stabilisation sufficiency and controlled sustained biting were observed. Established clinical signs of aspiration such as increased respiratory rate, audible breathing, shallow breathing, coughing, throat clearing, drooling, watering eyes, ‘wet’ vocalisation or choking (Arvedson et al., 2020) after pharyngeal swallow initiation were recorded.

#### The Rossetti Infant-Toddler Language Scale

Communication interaction, including pre-verbal and verbal skills, was assessed with the criterion-referenced Rossetti Infant-Toddler Language Scale (RITLS) (Rossetti, 2006). The comprehensive tool was previously used with a South African population (Van der Linde et al., 2015) and is appropriate for children from birth to 36 months. The RITLS identifies different developmental levels in 3-month intervals across the pragmatics, gesture, play, language comprehension, language expression and interaction-attachment domains. Because of health and safety restrictions in the hospital during the COVID-19 pandemic, the researcher relied on observation to identify communication interaction skills. Parental reports and elicitation of communication interaction with the same set of toys were supplemented only when required.

### Analysis

Information from the structured background information form was summarised descriptively and relevant data were tabulated. As per the SOMA protocol and scoring criteria, the researcher rated the swallowing behaviours of the second trial (bite) of each consistency as this is considered a more reliable presentation of oral-motor skills and swallowing behaviour (Reilly et al., 1995). A cut-off score was used to categorise the behaviours per consistency as functional or oral-motor dysfunction. Furthermore, oral-sensorimotor skills were considered across consistencies as well as discrete oral-sensorimotor behaviours for feeding.

Communication skills in each domain of the RITLS were evaluated across 3-month intervals, working backwards from the participants’ age intervals to establish their developmental levels (when all skills are performed at that interval). If no developmental level was identified, existing skills were considered emerging. If the established developmental level of any domain is two intervals or more below the chronological age, the participant was considered to present with a communication delay categorised as mild (6 months), moderate (6–12 months) or severe (12–15 months). According to RITLS guidelines (Rossetti, 2006), typical language development is attributed to achievement of skill levels 3 months below or above the expected level of functioning for the participants’ age. The global basal communication level is interpreted as the highest level where all items of each domain are achieved.

### Ethical considerations

Approval to conduct the study and collect the data was obtained from the University of Pretoria, Faculty of Humanities, Research Ethics Committee, reference number: HUM009/0320 and the Kalafong Provincial Tertiary Hospital (KPTH), reference number: KPTH 17/2020. Voluntary informed consent was obtained from mothers (≥ 18 years) to peruse medical files, conduct interviews and assess the toddlers. Mothers were aware that they could refute questions and/or withdraw consent anytime without elucidation.

## Results

### Feeding history

None of the participants received neonatal intensive care or tube feeding after birth. All had average birth weight and breastfed immediately. Participant 2’s mother reported pain at the start of BF, which resolved when nursing staff proposed position changes during feeding. Table 4 summarises the reported past and current feeding practices per case and maternal perceptions including reasons all mothers stopped exclusive BF. All mothers reported that participants’ feeding currently influences the family at mealtimes negatively because of disruptive feeding behaviours, which include food refusal, avoidance and moving away, slow eating, and use of distraction, and force-feeding. Participants had two-to-three meals on average per day. No additional snacks were reported, indicating daily intake being inadequate for nutritional requirements to support typical child development. The dietician noted that all participants experience limited dietary diversity at home.

**TABLE 4:** Reported feeding practices and maternal perceptions per case (*n* = 3).

Feeding practices	Case 1	Case 2	Case 3
**Breastfeeding (BF)**			
Exclusive BF	6 months	3 months	0 (2 weeks)
Age stopped direct BF	9 months	11 months	2 months
Reason stopped BF	Thought breastmilk made toddler sick.	Started using formula more and introduced solids.	Started giving water and returned to work.
Used anti-retrovirals while BF	Yes	Yes	Defaulted
**Bottle-feeding**			
Started bottle-feed formula	9 months	3 months	2 months
Current bottle-feeding	Yes	Yes	Yes
**Complementary feeding (CF)**			
Started CF	6 months	3 months	4 months
**Current duration per feed**			
Bottle-feed duration	30 min	40 min	50 min
CF duration	50 min	45 min	75 min
**Perception of duration of feeds**	Too short	Too short	Normal
**Daily feeds at home**			
Feeding frequency	3 scheduled meals 5 bottles	2 scheduled meals 5 bottles	2 scheduled meals 6 bottles
Number different CF per day	4	3	3
Give same CF twice per day	Yes	Yes	Yes
Where child eats at home	On mother’s lap	On floor and walking around	On grandmother’s lap
**Concern for toddler’s eating**	Worried	Not worried	Very worried
**Perception of toddler’s appetite**	Normal	Small	Large
**Food refusal**	At beginning	In middle	At beginning
**Use of distraction to feed**	Sometimes	Yes	Yes
**Follow child around**	No	Yes	Sometimes
**Give sweets to child**	Yes	Yes	Yes
**Use of force to feed**	Sometimes	Sometimes	Yes
**Mealtimes influence on family**	Negatively	Negatively	Negatively

CF: Complementary feeding (such as porridge, *Weetbix*, pap, bread, butternut, potatoes, meat and yoghurt).

### Swallowing characteristics: Schedule for oral motor assessment

During the SOMA, and confirmed by an independent physiotherapy assessment, it was observed that Participant 1 does not have sufficient postural control to sit independently (Table 1), indicating a severe gross motor developmental delay. Participants 1 and 3 reclined on their mother’s lap for support to feed while Participant 2 sat independently throughout most of the evaluation. Arvedson et al. (2020) describe that toddlers who are 13–18 months old should sit or stand independently to eat, predominantly self-feed, swallow with lip closure, demonstrate lateral and vertical tongue movements and gather food into a bolus with their tongue. Appropriate lip movement with rotary chewing should be used with no anterior food loss and food should be cleared off lips independently (Arvedson et al., 2020). Furthermore, all food consistencies should be tolerated. The participants in this study were all diagnosed with heterogeneous oral-sensorimotor dysfunction affecting food acceptance, bolus preparation, formation and propulsion, across a variety of consistencies (Table 5). All mothers were observed to clean participants’ lips and face frequently during feeding.

**TABLE 5:** Observed feeding skills and behaviours according to the schedule for oral motor assessment per case (*n* = 3).

Feeding skills	Case 1	Case 2	Case 3
**Oral-sensorimotor skills**			
Oral preparatory phase			
Head orientation to spoon	Yes	Yes	Yes
Initiated reaction for food acceptance	Yes	Yes	No
Graded jaw opening	No	No	No
Upper lip – removal of food from spoon	No	Yes	Yes
Lower lip – removal of food from spoon	No	No	No
Small vertical excursions	No	No	No
Internal jaw stabilisation	Yes	Variable	Variable
Sustained bite	No	No	No
Tongue lateralisation	Yes	No	Yes
Absence of drooling	No	No	No
Absence food pocketing	No	Yes	No
Pharyngeal phase			
Smooth swallow sequence	Yes	Variable	No
Absence of tongue protrusion	Yes	No	No
Lip closure during swallow	No	Yes	Yes
Absence anterior food/liquid loss	No	Yes	No
Absence multiple swallows	No	No	No
**Classification**			
Liquid – bottle	Functional	Functional	Functional
Liquid – cup	Functional	Functional	OMD
Puree	OMD	OMD	Functional
Semi-solid	Functional	OMD	Functional
Solid	OMD	OMD	OMD
Cracker	OMD	OMD	OMD
**Feeding behaviours**	Food refusal by head turn and holding mouth closed Holding food in mouth Use of distraction Use of forcefeeding	Use of distraction Use of forcefeeding at end Following toddler	Refusal by head turns, moving away Holding food in mouth Use of distraction Use of forcefeeding Following toddler

OMD, Oral-motor dysfunction.

Despite intact pharyngeal swallow responses, all participants presented with oral-sensorimotor dysfunction in the oral preparatory phase of swallowing. Difficulties with jaw opening and stabilisation, food removal with lips (particularly lower lip), sustained bite, tongue protrusion and lateralisation, bolus manipulation and mastication were observed. Poor lip closure was observed during the pharyngeal phase of swallowing. Participant 3 presented with oral-sensorimotor dysfunction when drinking from an open cup. Difficulties included the absence of lower lip support to the cup resulting in uncoordinated swallows, anterior spillage, multiple swallows and the observable reaction of panic when milk enters the mouth. Participants 1 and 3 presented with oral-sensorimotor dysfunction when eating purees and semi-solids, showing insufficient jaw opening, difficulty with regular chewing actions, tongue protrusion, anterior food loss and pocketing. Participant 1 presented with a single cough and Participant 3 presented with wet vocalisation soon after initiation of the pharyngeal swallow response with semi-solids, suggesting risk for aspiration.

Participants 1 and 3 swallowed small butternut cubes whole. All participants presented with oral-sensorimotor dysfunction in the oral preparatory phase when eating a cracker. Participant 2 showed poorly graded jaw opening, insufficient strength to sustain the bite, anterior drooling, sucking and reliance on the cracker getting soft, difficulty lateralising tongue, poor lip control and rotary movements of the jaw during chewing and severe pocketing in the buccal sulci. In the pharyngeal phase, multiple swallows and anterior food loss were noted. Mothers reported drooling onset between 1 and 3 months before hospital admission. During bedside assessment, drooling of mild-moderate severity was identified with all participants, particularly during feeding. This suggests poor lip closure, which may be associated with fatigue, limited endurance or delayed motor skills. Participants’ observed disruptive feeding behaviours included food refusal, holding food in their mouth, slow eating, and combinations of distraction and force-feeding by the mothers.

### Communication characteristics: Rossetti Infant-Toddler Language Scale

The RITLS showed that all three participants experience mild-to-moderate receptive-expressive language delay (Table 6). Participants had the most difficulty with interaction-attachment, gestures, play and language comprehension. Mothers of Participants 2 and 3 explained that their children do not say many words because they are sick and ‘too small’. Mothers of the participants reported that no newborn hearing screening was received. Participants 1 and 3 reportedly experienced recurrent middle ear infections; however, they were discharged before hearing screening could be conducted.

**TABLE 6:** Rossetti Infant-Toddler Language Scale results per case (*n* = 3).

Communication skills	Case 1	Case 2	Case 3
**Interaction-attachment**			
Interval achieved (months)	3–6	9–12	3–6
Delay (months)	9	6	8
Classification	Moderate	Mild	Moderate
**Pragmatics**			
Interval achieved (months)	3–6	12–15	6–9
Delay (months)	9	3	5
Classification	Moderate	To monitor	Mild
**Gesture**			
Interval achieved (months)	Emerging	9–12	Emerging
Delay (months)	15	6	14
Classification	Severe	Mild	Severe
**Play**			
Interval achieved (months)	3–6	9–12	3–6
Delay (months)	9	6	8
Classification	Moderate	Mild	Moderate
**Language comprehension**			
Interval achieved (months)	3–6	6–9	3–6
Delay (months)	9	9	8
Classification	Moderate	Moderate	Moderate
**Language-expression**			
Interval achieved (months)	6–9	9–12	6–9
Delay (months)	6	6	5
Classification	Mild	Mild	Mild
**Global basal communication level**			
Interval achieved (months)	3–6	6–9	3–6
Delay (months)	9	9	8
Classification	Moderate	Moderate	Moderate

## Discussion

This exploratory study provides a detailed description of swallowing, oral-sensorimotor function and communication development in a collective case-study of three hospitalised toddlers independently diagnosed with SAM. The description is relevant for SLTs practising in early communication intervention as well as the multi-disciplinary team. The toddlers presented with a triad of SAM, HIV and TB co-infection. Because of multiple data collection tools, a rich data set for each case could be described. Findings of heterogeneous oral-sensorimotor difficulties were identified with graded jaw opening, stabilisation, food removal, accurate tongue placement, lateralisation, and elevation for bolus manipulation, and sustained bite in the oral phase. Additionally, poor lip closure with multiple swallows was observed in the pharyngeal phase of swallowing indicating a need for dietary consistency modifications. Clinical signs of aspiration during the SOMA suggest risk for OPD. Disruptive feeding behaviours were also observed. Mild-to-moderate language delays were identified in all domains of the RITLS for all participants – except Participant 2’s pragmatic skills, which should be monitored. An atypical receptive-expressive language delay profile is highlighted, with moderately delayed language comprehension and mild expressive language delay only (Owens, 2020).

### Background, feeding history and developmental considerations

Outstanding information available in the case histories of all participants was their diagnosis of HIV at their initial hospital admission as well as TB co-infection. Prior South African research demonstrated that 54% of children in the sample were diagnosed with HIV at hospital admission (De Maayer & Saloojee, 2011). The late HIV-diagnosis is notable considering recent advances in South Africa’s Prevention of Mother-to-Child Transmission (PMTCT) campaign at clinics. Prevention of Mother-to-Child Transmission failure in participants may be attributed to social-behavioural strategies for the COVID-19 pandemic as the need for social distancing, transport difficulties and stigma led to avoidance of health facilities (Hofman & Madhi, 2020; Nyashanu et al., 2020). Active TB and HIV, previously managed at clinics, were diagnosed for all participants during hospital stay. This delayed identification further highlights how COVID-19 restrictions disrupted essential screenings at clinics to address South Africa’s quadruple burden of disease (Hofman & Madhi, 2020). The effects of disrupted maternal and child healthcare services are further underscored as mothers of Participants 1 and 3 missed immunisations and baby wellness appointments, which may have identified poor anthropometry earlier.

Although participants were initiated on BF, they were not currently breastfed. Participants 2 and 3 stopped BF before 6 months old. Short exclusive BF duration and suboptimal dietary diversity are known risks for SAM, which may have played a role in participants’ poor anthropometry and nutrition (Akombi et al., 2017; Chopra, 2003). Because research with HIV-exposed-uninfected infants shows that appropriate BF practices can modify poor infant growth associated with maternal risk factors (Ndiaye et al., 2021), the low adherence to BF recommendations is concerning. Improved clinic attendance may have prevented early BF cessation and increased dietary diversity through maternal education.

The identified poor feeding practices are compounded by social risks including limited access to healthcare, hygiene and sanitation, and low maternal education and literacy, along with HIV and TB co-infection. These risk factors are congruent with established risks for undernutrition in South Africa as well as SAM internationally (Chopra, 2003; Lesiapeto et al., 2010; WHO & UNICEF, 2009). Although risk factors for undernutrition are identified, it is important to understand how biological and environmental risks contribute to precipitating and perpetuating factors of SAM in South Africa’s context with quadruple burden of disease. Further research is required to understand the interplay of mother and child individual health, burden of HIV and TB co-infection, and household socio-economic conditions, placing young children at risk of SAM in South Africa.

Delayed gross-motor skills of Participants 1 and 3 resulted in poor postural control and alignment for feeding, contributing to coughing, wet vocalisations and a risk for aspiration (Arvedson et al., 2020). Prior research with wasted children (WHZ < -2) and children with SAM (WHZ < -3) highlight motor delays, hypotonicity, limited endurance and observed disruptive feeding behaviours congruent with the current findings (Saldan, Demario, Brecailo, Ferriani, & Mello, 2015; Sudfeld et al., 2015). Potentially progressive motor delays can limit environment exploration and inhibit sensory, cognitive and language stimulation (Prado & Dewey, 2014). The findings outline the importance of SLT collaboration in the paediatric MDT (South African Speech-Language-Hearing Association [SASLHA], 2017) for the management of young children with SAM’s swallowing, feeding and communication development.

### Swallowing characteristics

The heterogeneous oral-sensorimotor dysfunction affecting controlled jaw opening, stabilisation, tongue placement and co-ordination, sustained bite in the oral phase and poor lip closure in the pharyngeal phase, highlights participants’ low muscle tone and weakness known to be associated with SAM (Arvedson et al., 2020). As seen in this study, increased respiratory effort due to TB reduces endurance, and may contribute to delayed initiation of swallow, extended pauses, reduced lip closure for oral inspiration and increased respiratory rate while feeding (Barbosa et al., 2014). Participants appeared to experience extended feeding times, increasing energy use, which may prolong nutritional recovery. The description of oral-sensorimotor dysfunction contributes novel information that toddlers with SAM are at risk for OPD after transitions to oral feeding and possible aspiration pneumonia, which may extend hospital stay (Dodrill & Gosa, 2015). The findings indicate the importance of continued research to inform the role of SLTs in early intervention for this vulnerable population during hospitalisation.

Anterior drooling was identified and may be related to poor oral muscle tone and co-ordination, OPD and/or open-mouth posture because of TB-related respiratory difficulties (Montgomery et al., 2016). These difficulties impact swallowing and speech development (Arvedson et al., 2020). Drooling is therefore a characteristic that health care professionals can observe as an indicator to refer for SLT assessment. Low muscle tone contributing to oral-sensorimotor dysfunction and drooling may be attributed to neurological changes in the brain resulting from SAM and HIV interactions, specifically impacting motor development and control (Arvedson et al., 2020; Bhargava & Singh, 2020; Ruiseñor-Escudero et al., 2016). Findings of oral-sensorimotor dysfunction and drooling indicate that risk for OPD was not identified as a contributing factor. Referral for specific SLT management is thus required.

The findings show that children with SAM, HIV and TB co-infection can experience oral feeding difficulties after acute medical treatment, thus indicating the importance of early SLT involvement. Speech-language therapists should provide earliest holistic swallowing and oral-sensorimotor intervention (Barbosa et al., 2014). In this collective case-study, none of the participants were referred for SLT assessment at the time of data collection. Consequently, oral-sensorimotor dysfunction contributing to feeding difficulties and disruptive feeding behaviours were not identified for early intervention. Because of high SLT caseloads children with SAM may not be seen timeously by an SLT during acute treatment. In tertiary hospitals where SLT services are readily available, SLTs should typically be involved to assess safety of swallowing and feeding when transitioning patients to oral feeding. Findings of this study show that late referral can result in missed opportunities to advance children’s development. Referral to SLT services for assessment and/or intervention should be included in SAM management guidelines (DOH, n.d.; WHO, 2013).

### Communication characteristics

All participants experienced mild-to-moderate language delays. Central nervous system viral infection as a result of HIV infection, as well as SAM (WHZ < -3) and wasting (WHZ < -2) in children impair cognitive, behavioural, language and motor development of young children who learn through play (Gómez et al., 2018; Ruiseñor-Escudero et al., 2016; Sudfeld et al., 2015). HIV infection significantly impairs development of executive function, working memory and recall for learning, thus impacting children’s abilities to acquire language knowledge (Ruiseñor-Escudero et al., 2016). Poor motor development and fatigue limit play, environmental exploration and interaction opportunities with caregivers to develop receptive language (Iannotti et al., 2016; Prado & Dewey, 2014). These limitations may contribute to the particpants’ uncommon profile of better expressive language skills. As a result of nutritional deficiency, toddlers may be functionally isolated. Toddlers may not initiate interactions to receive language input and mothers initiate less because of toddlers’ lack of responsiveness (East et al., 2019). Early communication intervention can successfully resolve functional isolation and repair mother-child interactions (Itaka & Omole, 2020). The pathophysiological effect of SAM and HIV on neurodevelopment likely exacerbated delays shown in interaction-attachment and play subtests of the RITLS through gross and fine-motor skills delay.

Recurrent middle ear infections are strongly associated with hearing loss and were reportedly experienced frequently by all participants, temporarily limiting their access to new sounds and language. Emerging research shows that children with SAM are predisposed to specific patterns of hearing loss, which may be temporary or permanent, with independent association of moderate-to-profound, more persistent, sensorineural and high-frequency hearing loss (Close et al., 2020). Future research should aim to describe hearing and middle ear functioning in prospective studies.

### Implications for early childhood intervention

Although SAM, HIV and/or TB are not new diagnoses for SLTs to address, this study highlights the complexity of their combined presentation in young children. Novel detailed descriptions of the swallowing, feeding and communication profiles of young children with SAM are presented. The profiles emphasise the need for early SLT intervention. Current standards of practice indicate that young children with uncomplicated SAM should be managed as out-patients in clinics to decentralise care. Distance to health facilities and the lack of transport remain an obstacle for continued follow-up and access to rehabilitative services for children with SAM (Hofman & Madhi, 2020; Nyashanu et al., 2020). Research shows that pervasive neurodevelopmental effects of SAM may be reversed through nutritional rehabilitation and effective stimulation (Bhargava & Singh, 2020). Findings underscore the need for paediatric rehabilitation specialists such as SLTs to maximise opportunities to provide early intervention and caregiver coaching while toddlers are still hospitalised.

Limitations of analysis include the cross-sectional nature of the study as some observed behaviours may change as recovery progresses. Longitudinal studies are therefore warranted. Clinical assessments and observations can be furthered with instrumental assessments in future studies. This research presents in-depth descriptions of three cases, but generalisation of findings to other toddlers with SAM is not possible. The exploratory nature of the investigation prevents determining associations between factors in these cases. Findings therefore indicate a need to further investigate the swallowing, oral-sensorimotor and communication development of young children hospitalised with SAM. Future research endeavours may support sustained nutritional recovery and language development using larger-scale experimental, longitudinal or case-control study designs. The cases presented are presumed to be commonly part of the SLT caseload in hospitals; therefore, further research is imperative.

## Conclusion

The complexity of SAM, HIV and TB co-infection was a notable characteristic in all participants’ case histories and their presenting symptoms. Despite nearing readiness for discharge to community-based management because of nutritional recovery (according to anthropometric measurements), participants in this collective case-study presented with oral-sensorimotor dysfunction and disruptive feeding behaviours. Moreover, an atypical receptive-expressive language delay profile is highlighted with moderately delayed language comprehension and mild expressive language delay only, warranting early communication intervention. However, the three toddlers were not referred for SLT services. The findings indicate that South African children with SAM and simultaneous HIV and TB co-infection may experience synergistic detrimental effects requiring highly co-ordinated early MDT intervention. The early involvement of the hospital-based SLT to support young children with SAM, before they are dispersed to communities with limited services, is important.
